# Determination of Gastric Water Emptying in Fasted and Fed State Conditions Using a Compression-Coated Tablet and Salivary Caffeine Kinetics

**DOI:** 10.3390/pharmaceutics15112584

**Published:** 2023-11-04

**Authors:** Theodora Tzakri, Lara Rehenbrock, Stefan Senekowitsch, Adrian Rump, Philipp Schick, Julius Krause, Marie-Luise Kromrey, Michael Grimm, Werner Weitschies

**Affiliations:** 1 Department of Biopharmaceutics and Pharmaceutical Technology, Center of Drug Absorption and Transport (C_DAT), University of Greifswald, Felix-Hausdorff-Str. 3, 17489 Greifswald, Germany; 2 Department of Diagnostic Radiology and Neuroradiology, University Medicine Greifswald, Ferdinand-Sauerbruch-Str., 17489 Greifswald, Germany

**Keywords:** compression-coated tablet, gastric emptying, salivary tracer technique, caffeine, fasted and fed state conditions, in vivo study

## Abstract

Because of the importance of gastric emptying for pharmacokinetics, numerous methods have been developed for its determination. One of the methods is the salivary tracer technique, which utilizes an ice capsule containing caffeine as a salivary tracer. Despite the ice capsule’s advantage in labeling ingested fluids with caffeine for subsequent salivary detection, its risk of premature melting before swallowing, and its complicated storage and preparation, limit its application, particularly in special populations (e.g., older people). For this reason, here, a compression-coated tablet was developed and validated against the ice capsule in a cross-over clinical trial. The two dosage forms were administered simultaneously to 12 volunteers in an upright position under fasted and fed state conditions. To distinguish the caffeine concentrations in saliva from each dosage form, regular type of caffeine (^12^C) was added to the tablet, while for the ice capsule ^13^C_3_ labelled caffeine was used. The salivary caffeine concentrations showed no statistically significant differences for the pharmacokinetic parameters t_max_ and AUC_0→60_ (*p* > 0.05). Thus, the new formulation is a useful tool for determining gastric emptying that can also be used in special populations.

## 1. Introduction

Oral drug delivery is the most common route of administration, because of its non-invasiveness, patient adherence and safety [1,2]. As orally administered drugs must pass through the stomach before being absorbed in the intestines, gastric emptying time is an important physiological factor influencing their absorption kinetics [3,4,5,6]. The emptying process for non-caloric liquids (i.e., water) is typically described by first-order kinetics. In young healthy adults it has been found that 240 mL of still water is emptied from the stomach within 15–45 min, in both fasted and fed conditions. Unaltered emptying under postprandial conditions is due to the phenomenon of *Magenstrasse*, which describes a “gastric route” formed inside the fed stomach that allows the rapid emptying of water into the small intestine, and consequently of the dissolved or suspended drug substance [7,8,9]. On the contrary, less is known for the gastric emptying of liquids in geriatric patients and older people [10].

Most commonly, gastric emptying is determined by the performance of imaging studies, such as an MRI, which has a high cost and requires specialized equipment, and scintigraphy with the additional risk of radiation exposure. Pharmacokinetic markers are among the well-established methods; however, they may require frequent blood sampling [11,12]. For older people and geriatric patients, more simplified methods are needed, as they can allow for an application of the method to a wider range of populations. For instance, the salivary tracer technique, previously developed by Sager et al., is a non-invasive method for the determination of gastric water emptying, using an ice capsule containing a caffeine solution, since caffeine is used as a salivary marker. The volunteer can effortlessly collect saliva samples himself, allowing repeated sample collection for estimation of gastric emptying time either for physiological evaluations or during pharmacokinetic studies [13].

However, the ice capsule exhibits some limitations, which hinder its broad application, such as its size (15 mm diameter) which makes it difficult to swallow, especially for patients who have swallowing difficulties [14,15]. Moreover, its room temperature instability can result in rapid melting before intake, or caffeine contamination of the oral cavity, if not swallowed immediately. It is also difficult to use it in a broad range of settings (e.g., geriatric wards and hospitals), as the ice capsules must be frozen during production and transport. All of these factors can subsequently lead to the failure of this method.

The goal of this study was to develop an alternative vehicle for rapid delivery of caffeine into the stomach content. The new vehicle should present the following properties: (1) It releases caffeine just as quickly as the ice capsule, and therefore leads to emptying of the caffeine to the duodenum with the ingested water. (2) It remains stable at room temperature and in the oral cavity avoiding salivary contamination with caffeine while intake. (3) It has a small and convenient size. (4) It is easier to produce, store and transport. As with ice capsules, it is important to choose a preparation method that ensures that caffeine is only present within the vehicle and to prevent caffeine migration to the surface during the production process and during storage. The new vehicle was designed as a fast disintegrating tablet; however, a mechanism to avoid contamination of the oral cavity with caffeine during administration was mandatory. Due to the typically small volumes of saliva in the oral cavity, even the smallest amounts of caffeine would cause oral contamination. Therefore, a film-coated tablet was ruled out as an option, as there would be the risk of the caffeine from the tablet cores dissolving during the coating process, and thus getting into the coating [16]. For this reason, a compression-coated tablet was developed to optimize the application of the salivary tracer technique. The compression-coated tablet consisted of an inner core containing caffeine and an outer layer to protect the oral cavity from caffeine contamination.

The tablet was validated against the ice capsule, in an open cross-over clinical study of two study arms, under fasted and fed conditions in accordance with the recommendations for bioavailability/bioequivalence (BA/BE) studies [17,18]. In order to determine whether the tablet could detect gastric water emptying, the two dosage forms were given simultaneously to volunteers in an upright position. To distinguish the salivary caffeine concentrations natural ^12^C-caffeine was added to the tablet, and stable isotope labelled ^13^C_3_-caffeine was added to the ice capsule. The use of a stable isotope to monitor the release and absorption of caffeine measured by caffeine concentrations in saliva has already been applied to a recent study of in vivo oral dosage form disintegration [19], since ^12^C- and ^13^C_3_-caffeine have similar pharmacokinetic profiles [20].

## 2. Materials and Methods

### 2.1. Study Materials

The composition of the compression-coated tablets and the respective quantities of the ingredients used are provided in Table 1 and Table 2. ^12^C-caffeine, saccharin sodium, Methocel E4M^®^ (hydroxypropyl methylcellulose) and black iron oxide were purchased from Caesar & Loretz GmbH (Hilden, Germany). Vivasol^®^ (croscarmellose sodium) and Prosolv^®^ (silicified microcrystalline cellulose) was obtained from JRS Pharma GmbH & Co. KG (Barsbüttel, Germany). Aerosil^®^ (amorphous colloidal silicon dioxide), Avicel^®^ (microcrystalline cellulose) and magnesium stearate were kindly gifted from F. Hoffmann-La Roche AG (Basel, Switzerland). ^13^C_3_-caffeine was purchased from Sigma-Aldrich Chemie GmbH (Schnelldorf, Germany). Aluminium bags used for packaging the tablets were purchased from Ströbel GmbH (Langenzenn, Germany). All solvents used for HPLC, i.e., water and methanol were of analytical grade, and all solvents used for LC-MS were of LC-MS grade.

### 2.2. Preparation of Core and Compression-Coated Tablets

For the core powder mixture, saccharin sodium was pulverized to achieve a fine powder using an Ultra-Centrifugal Mill ZM100 (Retsch, Haan, Germany) at 14,000 rpm with a 500 μm mesh width. All components, except magnesium stearate, were sieved through a 500 μm sieve, filled into a brown glass bottle of 0.5 L capacity and subsequently mixed for 5 min with a TURBULA^®^ 3D powder blender mixer (Willy A. Bachofen AG, Muttenz, Switzerland) at 49 rpm. Afterwards, the mixed powder was divided into two equal parts and magnesium stearate was added to the first half of the powder after sieving through an 800 μm sieve. The second half of the mixed powder was added, and the final powder mixture was mixed with the blender mixer for 1 min. Round flat-faced shaped core tablets of 5 mm diameter were then compressed using a Nagema KP2 eccentric tablet press (VEB Kombinat Nagema, Dresden, Germany). Each core tablet had a mass of approximately 50 mg corresponding to 25 mg of caffeine per tablet.

For the outer layer powder mixture, Prosolv^®^ and Methocel E4M^®^ were weighed and sieved through a 500 μm sieve filled into a white glass bottle of 1 L capacity, and then mixed for 5 min with the blend mixer at 49 rpm. Magnesium stearate was sieved through an 800 μm sieve, and then was incorporated into the Prosolv^®^-Methocel E4M^®^ mixture. The final powder mixture was further mixed for 1 min. The compression-coated tablets were pressed with the eccentric tablet press using biconvex-shaped punches of 9 mm diameter. About 110 mg of the outer layer powder was filled manually into the die cavity and its surface was flattened. Then, one core tablet was carefully placed in the middle of the powder surface. Afterward, the lower punch was placed at its lowest level and another 110 mg of the outer layer powder was filled into the die cavity to create the upper part of the tablet. To ensure accurate mass uniformity of the final formulation, the powder surface was flattened before compressing. After the batch production, the tablets were packaged individually into aluminium bags, which were heat-sealed.

### 2.3. Evaluation of Physical Properties

The cores as well as the compression-coated tablets were examined for their physical properties like mass uniformity, height, hardness, friability and disintegration time. For the assessment of mass uniformity, 20 tablets of each formulation were weighed using an electronic weighing balance (Sartorius Lab Instruments GmbH & Co. KG, Göttingen, Germany). The heights of the tablets were measured with a digital caliper (Burg-Wächter KG, Wetter, Germany). The hardness of 10 tablets was evaluated using a tablet hardness tester (Erweka GmbH, Langen, Germany). Friability was determined by weighing approximately 6.5 g of tablets, and then placing them in a friabilator equipped with a rotating drum (Erweka GmbH, Langen, Germany). The drum was rotated for 4 min at 25 rpm. Then, the tablets were dedusted and reweighed to determinate the % weight loss. Disintegration time was determined using a disintegration apparatus Erweka ZT 222 (Erweka GmbH, Langen, Germany). Six compression-coated tablets were tested simultaneously in 600 mL of distilled water at 37 ± 2 °C, with an upward and downward movement of from 29 to 32 times per min. Additionally, disintegration fluted discs were used to ensure the submersion of the tablets into the medium. The maximum acceptable disintegration time was set at 60 s to ensure that the tablet disintegrates fast enough to label the ingested water.

### 2.4. Content Uniformity

The monograph 2.9.40. of the European Pharmacopoeia was applied to determine the average content of ten randomly selected compression-coated tablets and the deviations of the individual contents [21]. To ensure that the samples were within the validated concentration range, each tablet was dissolved in a 100 mL volumetric flask with SGF_sp_ (simulated gastric fluid without pepsin of pH 1.2). Then, 200 µL of the solution was transferred into an Eppendorf Tube^®^, supplemented with 800 µL SGF_sp_, vortexed for one min, and then centrifuged at 13000 rpm for ten min. The supernatant was transferred to vials and used for caffeine quantification by an UHPLC-PDA System (Shimadzu Corporation, Kyoto, Japan). Caffeine was separated by gradient elution with 10 mM phosphate buffer of pH 6.8 and methanol as mobile phase. A flow rate of 0.4 mL/min was used. A Kinetex^®^ core-shell F5 column (2.6 µm, 2.1 × 150 mm, 100 Å; Phenomenex, Torrance, CA, USA) equipped with a SecurityGuard^TM^ ULTRA Cartridge (Phenomenex, USA), which was connected to a SecurityGuard^TM^ ULTRA Holder (Phenomenex, USA), was used as the stationary phase. The temperature of the column oven was set to 45 °C and the injection volume was set to 10 μL. The detection wavelength of caffeine was 272 nm and the retention time was 6.8 min. The LabSolutions software (Version 5.93, Shimadzu Corporation, Kyoto, Japan) was used to evaluate the chromatograms using the peak-area ratios (linear regression, 1/x^2^ weighting). The analytical method was validated in terms of linearity, precision (within-run and between-run), accuracy (within-run and between-run), selectivity, working range, freeze and thaw stability and rack stability according to the ICH Guideline Q2 (R1) of validation of analytical procedures [22]. The lower limit of quantification (LLOQ) was 5 μg/mL.

### 2.5. In Vitro Dissolution Study

The release of caffeine from the tablets was investigated using a Pharma Test DT 70 USP II paddle apparatus (Erweka GmbH, Langen, Germany). To simulate worst-case conditions, the experiments were performed at 25 °C, a stirring speed of 25 rpm and 300 mL of SGF_sp_ (pH 1.2) as dissolution medium, following the same protocol as Sager et al. [13]. Initially, the drug release was carried out for 10 min, and 2 mL of the sample was drawn every 30 s using a sample extractor and syringes. After every withdrawal, 2 mL of SGF_sp_ was added so that the volume of the medium remained constant during the release. The caffeine concentration was measured by the same validated UHPLC-PDA method.

### 2.6. Stability Studies

The stability study of the tablets was designed according to the ICH (Q1C) guidelines of stability testing for new dosage forms [23]. The packaged tablets were stored for three months under accelerated stability conditions (40 °C and 75% relative humidity) and under long-term stability conditions (25 °C and 60% relative humidity) right after their production. The stability was investigated by comparing the subsequent caffeine release of the tablets before and after storage.

### 2.7. Preparation of the Ice Capsules

The ice capsules were produced according to Sager et al. [13]. Each ice capsule contained approximately 25 mg ^13^C_3_ labelled caffeine and 250 mg saccharin sodium. The content uniformity and in vitro dissolution release of the ice capsules were tested in a paddle apparatus using the same conditions as for the compression-coated tablets.

### 2.8. Cross-Over Clinical Trial

**Figure 1:**
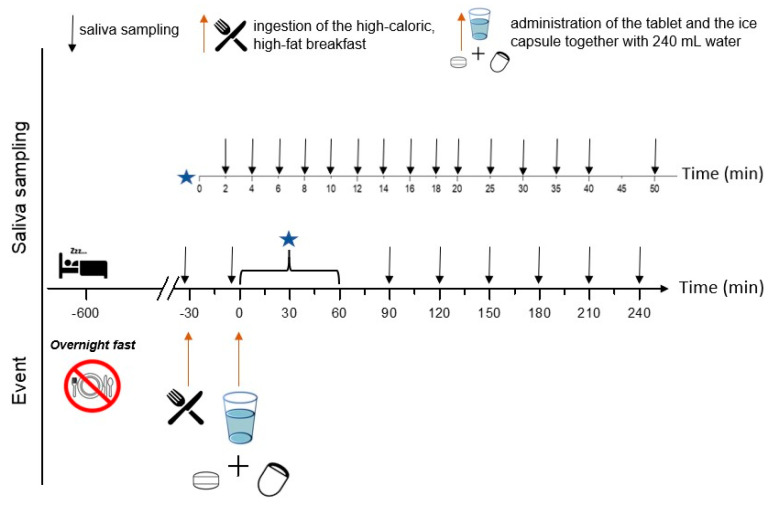
Timeline of the study day with specific events and saliva sampling time points for both study arms. The fasted state arm did not include meal ingestion. The star refers to the time segment from 0 to 60 min.

This study was performed at the Clinical Research Unit of the Department of Clinical Pharmacology, University Medicine Greifswald, in compliance with the Declaration of Helsinki (2013, Fortaleza, Brazil) and the “(Model) Professional Code for Physicians in Germany” (amended 2015 in Frankfurt, Germany). The study protocol was approved by the ethics committee of the University Medicine Greifswald, Germany (ethical protocol No. BB 220/21). The study has been registered at the German Clinical Trials Register (Deutsches Register Klinischer Studien) under the ID: DRKS00032457. The study met all bioethical regulations of the European ITN AGePOP project. Every subject was provided insurance to cover commuting accidents and risks arising from participation in the study. The exact timeline of the study can be seen in Figure 1.

The clinical study was conducted in an open cross-over design with two study arms. Under fasted state conditions, volunteers ingested the two dosage forms together with 240 mL of water, after an overnight fast of at least 10 h. Under fed state conditions, volunteers consumed a high-caloric, high-fat standard FDA breakfast 30 min before the administration of the dosage forms with 240 mL water. The breakfast consisted of two slices of toasted bread (Sammy’s Super Sandwich, Harry-Brot GmbH, Schenefeld, Germany), 40 g butter (Meggle Alpen-butter Minipack, Meggle AG, Wasserburg am Inn, Germany), two strips bacon (Tulip Bacon, Tulip Food Company, Randers, Denmark), 113 g hashed brown potatoes (Rösti-Ecken, Gut & Günstig EDEKA Group, Hamburg, Germany), two fried eggs (Frische Eier aus Bodenhaltung, ja!, REWE, Cologne, Germany) and 240 mL of full-fat milk (Haltbare Milch 3.5%, Weihenstephan, Germany). The total caloric content of the breakfast was approximately 1000 kcal.

### 2.9. Volunteers

Twelve healthy volunteers (7 female and 5 male) with a mean age of 25 ± 2 years (range 22–31 years) and with a mean BMI of 23 ± 3 kg/m^2^ (range 18.83–29.05 kg/m^2^) were recruited. Written informed consent was obtained from all participants before the beginning of the study to consent for handling of personal data and confirm German laws of data protection. The inclusion criteria were as follows: no history of bowel or GI tract disease or surgery, no heavy caffeine drinkers or heavy smokers, and no intake of medications affecting gastrointestinal motility or gastric pH. During the whole period of the study, no medications were allowed except for oral contraceptives. Volunteers had to abstain from the consumption of products containing caffeine at least 72 h before the start of the study day. In addition, the volunteers were asked to avoid any strenuous physical activity and alcohol consumption 24 h before the begin of the study day.

### 2.10. Saliva Sampling

The volunteers gave saliva samples by spitting directly into 1.5 mL SafeSeal microtubes (Sarstedt, Nümbrecht, Germany) within 20 s, at predetermined timepoints. At least 500 μL saliva was demanded and no artificial saliva stimulation was allowed. Blank samples were collected prior to intake of the dosage forms and the FDA breakfast to ensure the absence of caffeine in saliva. After the intake of the dosage forms (t = 0 min) saliva samples were collected for 240 min, and then stored at −80 °C until analysis.

### 2.11. Sample Preparation and Analysis

The samples were thawed at room temperature for approximately 60 min before preparation, then vortexed for 1 min and centrifugated for 10 min at 13,000 rpm with a microcentrifuge (Biofuge^®^ Pico, Heraeus, Hanau, Germany). The saliva sample preparation was performed according to the method of Senekowitsch et al. [24].

Determination of caffeine in the saliva samples was performed using a LC-MS 8060 Triple Quadrupole System (Shimadzu Corporation, Kyoto, Japan). The mass spectrometer processed with electro spray ionization (ESI) in positive multiple reaction monitoring mode. The mass transition for ^13^C_3_-caffeine were 198.20 → 140.10 (collision energy −20.0 V) and for ^12^C-caffeine were 194.90 → 138.05 (collision energy −20.0 V). Caffeine was separated by gradient elution with water (containing 1% formic acid) and methanol as mobile phase. A flow rate of 0.4 mL/min was used. A Kinetex^®^ reverse phase C_18_ column (2.6 µm, 2.1 × 150 mm, Phenomenex, USA) protected by a SecurityGuard™ ULTRA Cartridge (Phenomenex, USA) which was connected to a SecurityGuard™ ULTRA Holder (Phenomenex, USA) was used as the stationary phase. The temperature of the column oven was set to 40 °C. The injection volume was 10 μL total; 5 µL of the prepared sample was co-injected with 5 µL of a mixture of water with 1% formic acid. The retention time of caffeine was 2.48 min. The LabSolutions software (Version 5.97 SP1, Shimadzu Corporation, Kyoto, Japan) was applied to evaluate the chromatograms using the internal standard method and peak-area ratios for calculation (linear regression, 1/x^2^ weighting). The analytical method was validated in terms of linearity, precision (within-run and between-run), accuracy (within-run and between-run), selectivity, freeze and thaw stability, short-term stability at room temperature (up to 48 h), long-term stability at −80 °C (up to 6 months) and rack stability regarding the EMA guidelines [25]. The lower limit of quantification (LLOQ) was 2 ng/mL for both ^12^C-caffeine and ^13^C_3_-caffeine in saliva.

### 2.12. Data Evaluation and Statistics

To compare the kinetic curves of the tablet and the ice capsule, salivary caffeine concentrations were normalized by setting C_max_ to 100% and the pharmacokinetic parameters t_max_ and normalized AUC_0→60 min_ were calculated. The method of normalization has been reported already in previous gastric emptying studies [13,26]. Implausible data points (e.g., due to caffeine contamination in the oral cavity, false signals due to errors in sample preparation or analytical method sensitivity) were excluded from analysis. The caffeine concentrations from blank saliva samples (t = −2 min and t = −32 min) were excluded from the analysis since they were used only to control whether caffeine was present in the saliva before the start of the study. Graphical representations were depicted with OriginPro 8.5.1 (OriginLab, Northampton, MA, USA). The statistical analysis was conducted using GraphPad Prism 5 (GraphPad Software Inc., La Jolla, CA, USA). Then, the parameters t_max_ and normalized AUC_0→60_ were tested for normal distribution using the Kolmogorov–Smirnov and the D’Agostino–Pearson omnibus normality tests. Differences between the tablet and the ice capsule were tested by a two-tailed paired *t*-test in case of a normal distribution or by Wilcoxon signed rank test, in case normal distribution could not be proven for at least one group of data. A *p*-value of <0.05 was considered to be significant. The linear relationship between the tablet and the ice capsule was defined by Pearson’s correlation coefficient (r) with *p* < 0.01 considered to be significant.

## 3. Results

### 3.1. Tablet Production and Physicochemical Characterization

**Figure 2:**
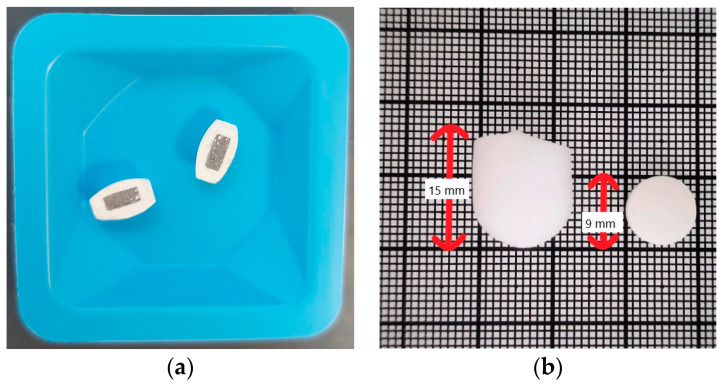
Exemplary images of the two formulations: (**a**) Compression-coated tablets cut in the middle; (**b**) size comparison of the ice capsule (**left**) and the compression-coated tablet (**right**) on millimetre paper.

The results of the physicochemical properties of the final formulation are presented in Table 3. The mass uniformity test of the core tablets showed that not more than two of the 20 cores weighed deviated 10% from the mean, which is the limit for tablets weighing less than 80 mg, with a range from 44.0 to 53.7 mg. For the compression-coated tablets, a deviation of less than 5% from the mean was achieved, with a range from 256.2 to 283.2 mg. The caffeine content was 100.1 ± 4.3%. The friability test showed a mass increase of 0.04%, lying within the analytical precision of the scale; however, no loss of mass could be measured. Representative images of the tablet and the ice capsule are shown in Figure 2.

### 3.2. In Vitro Dissolution and Stability Studies

**Figure 3:**
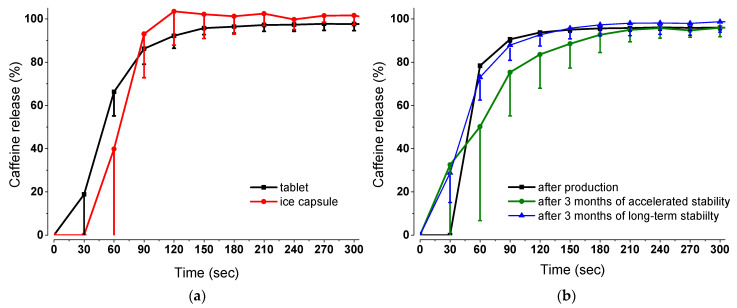
Dissolution profiles over time in 300 mL SGF_sp_ pH 1.2 at 25 ᵒC and 25 rpm: (**a**) Comparison of the caffeine release of the tablet and the ice capsule (*n* = 3, mean—standard deviation); (**b**) caffeine release of the tablets after 3 months of stability studies (*n* = 3, mean—standard deviation).

The caffeine release profiles of the two dosage forms are shown in Figure 3a. The increase in the mean caffeine release of the tablet started after 30 s, whereas that of the ice capsule started after 60 s. It can be seen that caffeine release from the tablets was faster at the beginning than from the ice capsules. The 85% caffeine release limit was used for comparability. After about 90 s, the two curves intersected at a release of about 85%, while the tablet reaches a release plateau at about 180 s. The maximum mean releases of the tablets and the ice capsules were 96.22% and 98.40%, respectively.

The results of the stability study after 3 months are represented in Figure 3b. Under long-term storage conditions, the tablets exhibit similar caffeine release kinetics to those tested on their production date. Under accelerated storage conditions, the release curve slightly decreased. Specifically, the time reaching 85% of the released fraction is within the range of 150–180 s in contrast to the production date, showing there is a delay of 90 s.

### 3.3. In Vivo Study

**Figure 4:**
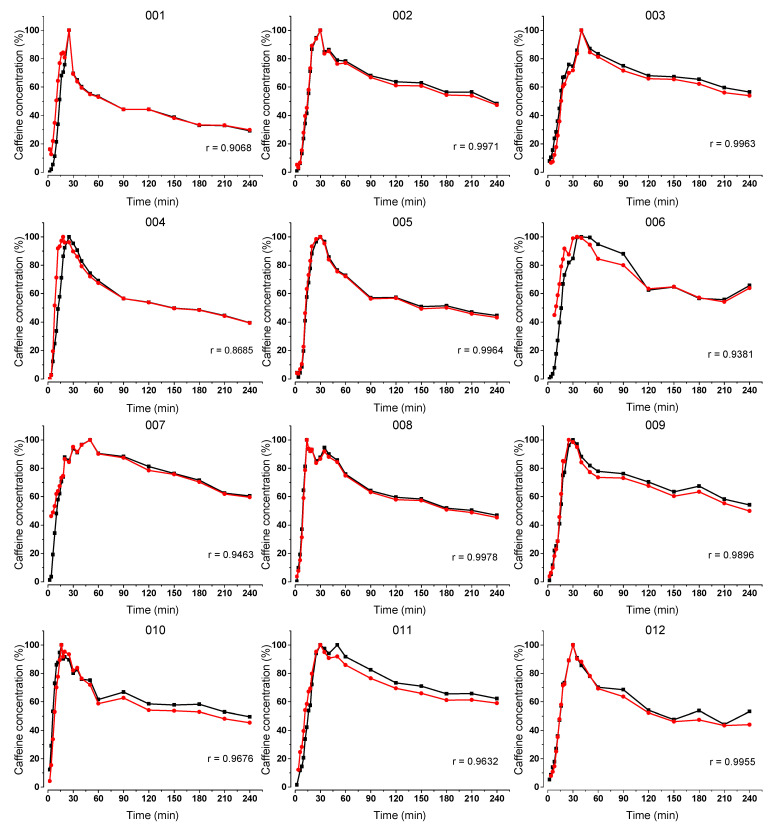
Normalized salivary caffeine concentrations of the compression-coated tablet (black) and the ice capsule (red) over time after their administration together with 240 mL tap water. Each graph shows data of each individual volunteer and the corresponding Pearson’s correlation coefficient (r).

**Figure 5:**
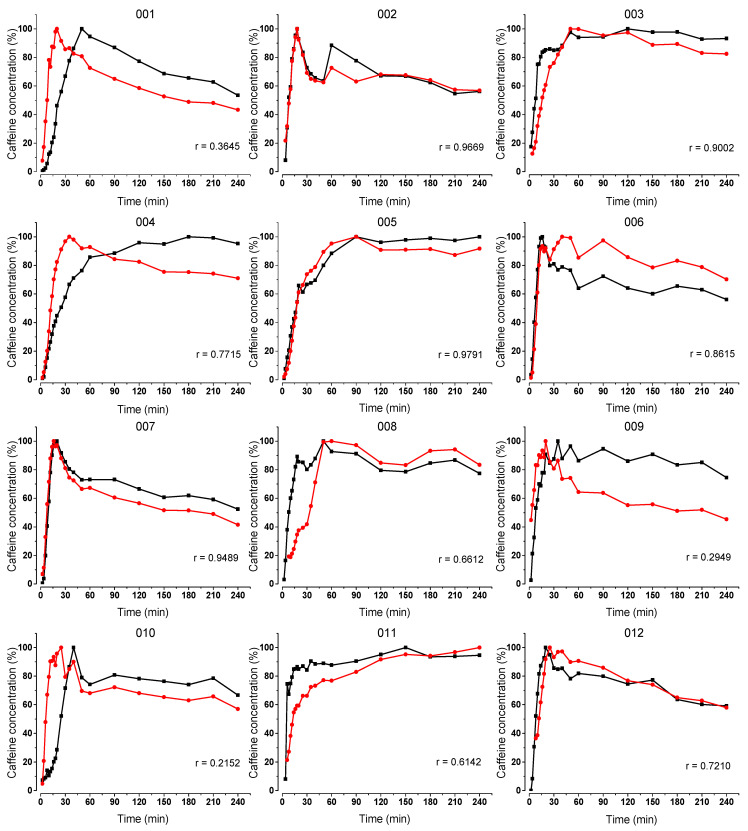
Normalized salivary caffeine concentrations of the compression-coated tablet (black) and the ice capsule (red) over time after their administration together with 240 mL tap water 30 min after intake of the FDA standard meal. Each graph shows data of each individual volunteer and the corresponding Pearson’s correlation coefficient (r).

All volunteers completed the study and tolerated all study procedures. Caffeine contamination by melting of the ice capsule in the oral cavity occurred in three volunteers (006, 008 and 011) and was observed as extremely high caffeine concentration on the first measurement time points. These data points were extracted from the statistical evaluation. Some volunteers failed to comply with the 72-h caffeine restriction, resulting in a higher onset of salivary caffeine concentration. The individual profiles of the salivary caffeine concentrations of the two dosage forms, as well as the corresponding Pearson’s correlation coefficient (r) can be found in Figure 4 for the fasted state and in Figure 5 for the fed state.

In fasted state, it can be seen that the initial increases in the salivary caffeine concentrations of the two formulations were similar for all of the 12 volunteers and the curves run parallel to each other throughout the whole duration of the study. Pearson’s correlation coefficient indicates a strong correlation for the 12 volunteers since the respective values are above 0.8 (*p* < 0.01).

In fed state, a higher variability can be observed. In some cases, the caffeine concentration of the ice capsules rises steeper and sometimes in reverse. The Pearson’s correlation coefficient indicated a moderate correlation for three volunteers (001, 009 and 010) since the respective values were below 0.5 (*p* < 0.01). Nevertheless, the initial increases in caffeine concentrations were similar for the tablet and the ice capsule in seven volunteers (002, 003, 005, 006, 007, 009, 011 and 012).

### 3.4. Comparison of the Compression-Coated Tablet and the Ice Capsule

**Figure 6:**
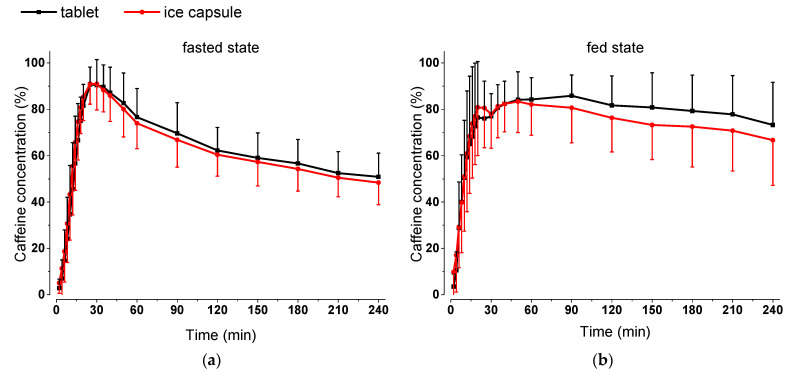
Mean curves of the normalized salivary caffeine concentrations (*n* = 12, mean +/− standard deviation) after administration of the two dosage forms in (**a**) fasted state and (**b**) fed state.

The mean curves of the normalized salivary caffeine concentrations are provided in Figure 6. As depicted in the graphs, in both fasted and fed state conditions, the kinetic curves of the two dosage forms run parallel to each other. The standard deviations indicate that caffeine concentrations from the fed state were more variable than those from the fasted state.

**Figure 7:**
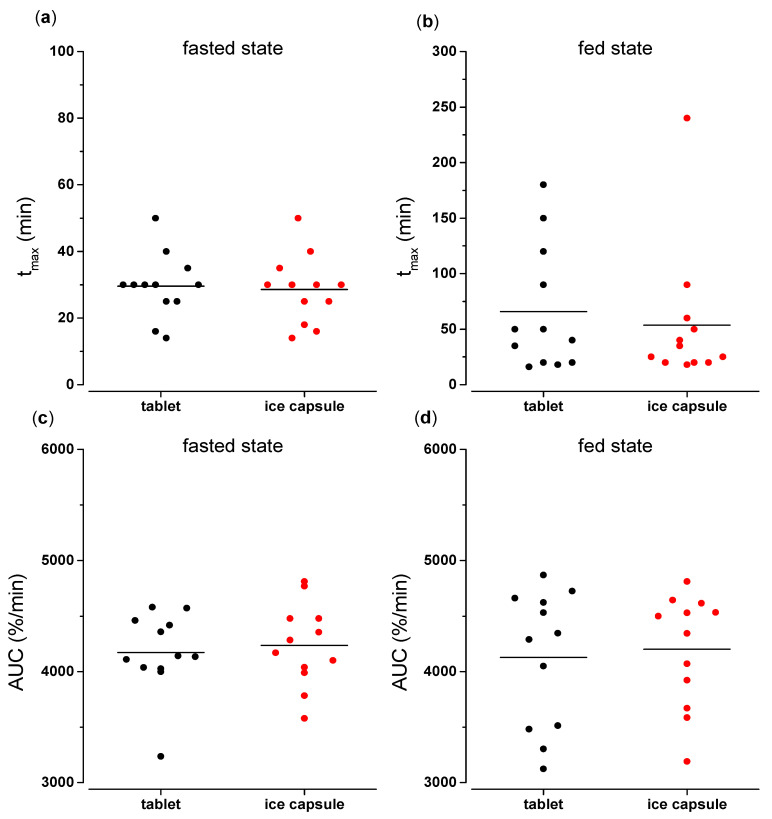
Distribution of t_max_ (min) and normalized AUC_0→60_ (%/min) after administration of the two dosage forms (tablet (black) and ice capsule (red)) with 240 mL water in fasted and fed state conditions (*n* = 12). The individual data are represented as points and the mean value is depicted as a straight line: (**a**) t_max_ in fasted state ranges, i.e., tablet, 14–50 min and ice capsule, 14–50 min (**b**) t_max_ in fed state ranges, i.e., tablet 16–150 min and ice capsule 18–240 min (**c**) AUC_0→60_ in fasted state ranges, i.e., tablet 3237–4580%/min and ice capsule 3579–4812%/min; (**d**) AUC_0→60_ in fed state ranges, i.e., tablet 3124–4869%/min and ice capsule 3190–4811%/min.

The mean values of the pharmacokinetic parameters t_max_ and AUC_0→60_, as well as their statistical evaluation, can be found on Table 4. No significant differences could be found for t_max_ and AUC_0→60_ between the tablet and the ice capsule either in fasted or fed state conditions (*p* > 0.05). The distribution of the individual values can be found in Figure 7.

## 4. Discussion

In this present study, a compression-coated tablet with caffeine was developed and validated against the existing ice capsule for application of the salivary tracer technique. The compression coating process, which is more complex than the usually applied film coating, was chosen for the production of the tablets. Compression coating ensures that no caffeine is released during the manufacturing process as it might occur during film coating [27]. Thereby, the surface of the tablets can be guaranteed to be free of caffeine and that oral contamination with caffeine during ingestion can be ruled out.

Furthermore, the tablets cores and the coating were manufactured using direct compression. Although direct compression is used above all for processing moisture-sensitive and heat-sensitive substances, it exhibits many advantages among other tablet manufacturing processes such as simplicity, cost-effectiveness and low labor input [28]. A direct compression process is suitable in this case, since it requires fewer additional processing steps and causes less material loss (compared to wet granulation, for example) which is particularly important with respect to the high costs of ^13^C_3_-caffeine. Additionally, another asset of critical importance for the goal of this study was that direct compression optimizes the disintegration of tablets, as each drug particle is released from the tablet mass and can rapidly dissolve in the dissolution medium [29]. Typically, compression-coated tablets are manufactured using a rotary tablet press machine [24]. Though in this study, a single-punch tablet press was more suitable for the development of the tablet formulation and the small-scale production.

During the study, three volunteers had caffeine contamination in the oral cavity from the ice capsules, since they rapidly melted if not swallowed immediately, whereas this was not the case with the tablets. Although the melting of the ice capsule in the mouth can be recognized by the characteristic sweet–bitter taste of saccharine sodium, not all the volunteers whose data showed caffeine contamination became aware of it. A possible explanation could be the diffusion of caffeine into the outer ice shell during storage.

The amount of 25 mg of caffeine used for the determination of gastric emptying is already below the usual dose of caffeine in caffeinated beverages, and thus should not raise concerns about its safety [30] or its pharmacological effect [31,32]. Similarly, in previous studies with the application of the salivary tracer technique, about 25–35 mg of caffeine were used [13,19,20]. Moreover, a recent study showed no influence of this caffeine labelling on gastric emptying by comparison of MRI data sets [20]. Black iron oxide was used to distinguish the core (black powder) from the coating (white powder) during manufacturing. Since the tablets were produced manually, color differentiation was crucial for ensuring that the core was positioned accurately in the middle of the tablet. Furthermore, black iron oxide can be used as a contrast agent in MRI studies, extending the use of the tablet to imaging studies in the future [33,34,35].

The physicochemical properties of the compression-coated tablets corresponded to the guidelines of the European Pharmacopoeia. There is a rule of thumb for the rough estimation of the required hardness, which is that it should be approximately ten times the tablet’s diameter [36]. In the case of the tablet cores, the diameter was 5 mm; therefore, the estimated hardness was 50 N. It was also not allowed to be too low because, during the manufacturing process of the final formulation, the core should remain completely intact so that no caffeine could enter the outer coating. However, the measured values were clearly below the estimated values and the lower hardness of the core (20 ± 3 N) was chosen deliberately so that the disintegration time was such that the tablet cores could disintegrate quickly. The diameter of the final tablet was 9 mm; therefore, the mean value of 91 ± 6 N is very close to the estimated favored value. The optimal properties for hardness were tested in preliminary testing by varying the compression pressure with a defined die size and powder composition. As a result, easy handling of the tablets was possible, and the final formulation could be packed in aluminum bags without breaking. Sufficient hardness was further proven by the friability experiments. Furthermore, as shown in Figure 3a, the in vitro dissolution of caffeine from the tablet was similar to the that of the ice capsule, as above 85% released substance was reached within 90 s. Thus, the data demonstrate that the tablet released caffeine rapidly enough to mark the ingested water, and thus detect gastric emptying. Of particular importance for the new vehicle is its stability and storage without the need of a freezer, such as in the case of the ice capsule. As shown in Figure 3b, after 3 months of long-term storage conditions, the caffeine dissolution rate remains the same. Even though in accelerated storage conditions, the 85% released fraction is reached after 150 s, this change in comparison with the results of the production date is actually minor. The caffeine dissolution after 150 s is, in our opinion, still rapid enough to determine gastric water emptying kinetics. Nevertheless, the dissolution conditions which were applied correspond to the worst-case scenario. These results show that the tablets are adequately protected in aluminium bags and can still be used up to three months from production date.

The time interval of 72 h caffeine abstinence between the study days was determined as a function of the elimination constant of caffeine [37]. However, in some volunteers, detectable caffeine concentrations were observed in the blank saliva samples for both formulations tested. In the case of ^12^C-caffeine, this resulted either from non-compliance with the caffeine fasting regulations or from a carryover effect. A carryover effect may occur when a volunteer is a “slow metabolizer”, which means a slower degradation of caffeine by the CYP1A2 enzyme and a longer period of time that it remains detectable in saliva [38,39,40]. In the case of labelled caffeine, only a carryover effect could occur, since this type of caffeine is not found in natural edible products. For example, in volunteers 003, 010 and 011, in the fasted and fed state arms, there were detectable 1^2^C-caffeine concentrations in blank saliva samples probably from the tablets of the previous study day, while for volunteers 003 and 010, this was the case for ^13^C_3_-caffeine from a previous ice capsule.

The caffeine kinetics obtained in the fasted state show that, after ingestion of the two dosage forms, the curves are comparable (Figure 6). This observation is also supported by the statistical analysis, as no significant differences were found for t_max_ and AUC_0→60 min_. (Table 4.). Considering Pearson’s correlation coefficient, there is a strong correlation with an r > 0.8 (*p* < 0.01) for all volunteers. In our study, t_max_ after intake of the tablet with 240 mL water was 30 ± 10 min; thus, it was consistent with published data on gastric emptying of the corresponding volume of water administered in clinical trials under fasted conditions. Based on previous MRI studies, it was shown that the gastric emptying of 240 mL of water can be observed within from 15 to 60 min [41], or within 45 min from the fasted stomach [42], though the interindividual variability of gastric water emptying indicates that the observed variability was in line with literature [41].

In the fed state condition, however, there is a higher variability of the pharmacokinetic parameters compared to the fasted state condition, as seen from the ranges in t_max_ and AUC_0→60_ (Figure 7). A possible explanation could be the localization of the dosage form within the fed stomach directly after intake, which could be explained by the fact that the two dosage forms have different dimensions and densities. The ice capsule is practically a frozen caffeine solution that can easily float on the gastric content, whereas the tablet is dispersed in a suspension into the ingested water after intake and could be more easily mixed with the chyme. We aimed for a rapid disintegration of either the ice capsule as well as the tablet near the *Magenstrasse* which can provide a shortcut for fluids along the stomach wall and allow caffeine to get emptied into the intestine quickly, representing water emptying. In contrast, if the dosage form has a less favorably localization and floats on top of the gastric contents in the fundus, it will likely remain there for a longer time [43]. Most likely, the problem is a distribution effect; if the dosage form is initially mixed with the chyme, a local drug cloud is more likely to form because the distribution is limited by the chyme viscosity [44,45]. In this way, caffeine cannot mark the water quickly enough, but instead is being emptied to the duodenum along with the chyme. In healthy young volunteers, food is emptied from the stomach slower than water at a rate of about 2–4 kcal/min [46]. Furthermore, this study was performed in an upright position, whereas previous water gastric emptying studies, either in fasted or fed state conditions, have been conducted in a supine position [13,20,26,41,42]. Previous studies in fasted state have shown that changes in posture can have an influence on the intragastric distribution of a drug within the stomach [47,48].

The statistical evaluation of t_max_ and AUC_0→60_ showed that there are no statistical differences between the two dosage forms in both fasted and fed state conditions (Table 4), meaning that the compression-coated tablet can be applied as an alternative formulation for the investigation of gastric water emptying. The new formulation exhibits numerous advantages. First of all, it makes it easier for the volunteer to handle and ingest the formulation because it remains stable and has a convenient size. Additionally, it facilitates the researcher himself, since the formulation can be produced easily, stored and transported without needing a freezer, which allows a more feasible and advantageous application to a wide range of clinical trial sites and a broader range of population (e.g., geriatric patients and bedridden patients). Finally, the new formulation practically eliminates the risk of caffeine contamination.

## 5. Conclusions

Within the framework of this work, it was possible to develop and validate a compression-coated tablet that is suitable for the detection of gastric water emptying with the implementation of the salivary tracer technique. The statistical evaluation showed no significant differences for the pharmacokinetic parameters, meaning that the new formulation can detect gastric water emptying in a comparable manner to the established ice capsule. This method can be used as an easy, efficient and cost-effective alternative to other methods used for the detection of gastric water emptying even within bioequivalence and bioavailability studies. It could be utilized not only with young volunteers, but also older people or critically ill patients, to whom other invasive methods are difficult to apply.

## Figures and Tables

**Table 1:** Composition of the core tablets according to their average mass (mg).

Ingredients	Quantity (mg)	Quantity (%)
Caffeine	24.01	49
Saccharine sodium	9.8	20
Avicel^®^ PH 102	10.29	21
Vivasol^®^	2.45	5
Black iron oxide	0.98	2
Aerosil^®^	0.98	2
Magnesium stearate	0.49	1
Total	49.00	100

**Table 2:** Composition of the outer layer coatings according to their average mass (mg).

Ingredients	Quantity (mg)	Quantity (%)
Prosolv^®^ SMCC HD 90	214.37	97
Methocel E4M^®^	4.42	2
Magnesium stearate	2.21	1
Total	221	100

**Table 3:** Results of tests performed to evaluate the physical properties of tablets according to the European Pharmacopoeia (mean value ± standard deviation).

Parameter	Compression-Coated Tablet (9 mm)	Core Tablet (5 mm)
Average mass (mg)	270 ± 4	49 ± 2
Height (mm)	5.2 ± 0.1	2.1 ± 0.1
Hardness (N)	90 ± 6	20 ± 3
Disintegration time	within 21 s	within 15 s

**Table 4:** Mean values, standard deviation and statistical evaluation of the T_max_ and AUC_0→60_ of the two dosage forms in fasted and fed state conditions. ^1^ Normal distribution of the data set—a paired two-tailed *t*-test was applied. ^2^ No normal distribution of the data set—Wilcoxon signed rank test was applied.

Parameter		Compression-Coated Tablet	Ice Capsule	*p*-Value
T_max_ (min)	fasted ^1^	30 ± 10	29 ± 10	0.1725
	fed ^2^	66 ± 56	54 ± 62	0.4768
AUC_0→60_ (%/min)	fasted ^2^	4173 ± 364	4236 ± 370	0.2815
	fed ^1^	4126 ± 615	4201 ± 509	0.7749

## Data Availability

Data are provided in this article.
